# Efficacy and Long-Term Remission Following Haploidentical HSCT for Therapy-Related Acute Myelomonocytic Leukemia with Plasmacytoid Dendritic Cells Post-FCR Therapy for CLL: A Case Report

**DOI:** 10.3390/jcm15041559

**Published:** 2026-02-16

**Authors:** Alina Camelia Catana, Lidia-Maria Mondoc, Maria-Gabriela Vladoiu, Zsofia Varady, Camelia Dobrea, Horia Mihail Sandu, Liliana Mocanu, Ariela Olteanu, Geanina Mera, Minodora Teodoru

**Affiliations:** 1 County Clinical Emergency Hospital Sibiu, 550245 Sibiu, Romania; 2 Faculty of Medicine Sibiu, Lucian Blaga University of Sibiu, Lucian Blaga, 2A, 550169 Sibiu, Romania; 3 Fundeni Clinical Insitute, 022328 Bucharest, Romania

**Keywords:** CLL, FCR, haplotransplant, pDC-AMLs

## Abstract

**Introduction:** Chronic lymphocytic leukemia (CLL) is a common adult leukemia often treated with fludarabine, cyclophosphamide, and rituximab (FCR). While effective, FCR can lead to therapy-related myeloid neoplasms (t-MN), including aggressive therapy-related acute myeloid leukemia (t-AML). Stem cell transplantation offers the best chance for long-term remission in these cases. Here, we report a rare case of t-AML with plasmacytoid dendritic cells (pDC-AML) developing after FCR treatment for CLL that was successfully treated with haplotransplantation. **Case Presentation:** A 57-year-old woman with CLL-B was treated with six cycles of FCR, achieving a complete response. Six years later, at age 63, she developed t-AML with a rare morphophenotypic subtype: acute myelomonocytic leukemia with plasmacytoid dendritic cells (pDC-AML) and monosomy 8. Diagnostic challenges included distinguishing this subtype from blastic plasmacytoid dendritic cell neoplasm (BPDCN). She was treated with high-dose cytarabine followed by haploidentical stem cell transplantation from her son. Haploidentical transplantation was prioritized due to the urgent clinical need in a patient with high-risk acute leukemia (therapy-related leukemia secondary to prior chemoimmunotherapy and failure to achieve complete remission following the standard 3 + 7 induction protocol). In this critical setting, the patient’s son was immediately available as an HLA-haploidentical donor. Prior to the performance of the haploidentical stem cell transplant from her son, no HLA-matched unrelated donor (MUD) could be identified. Another viable alternative would have been the utilization of umbilical cord blood-derived stem cells harvested from the patient’s twin granddaughters. She was closely monitored post-transplant for potential complications, including graft-versus-host disease (GVHD), post-transplant lymphoproliferative disorder, and thyroid dysfunction, all of which were ruled out during follow-up. The patient remains in complete remission 15 years after her initial CLL diagnosis and 8 years after the t-AML diagnosis and haplotransplantation. Notably, no residual CLL clone was detected at the time of t-AML development, and a benign polyclonal lymphocytosis observed between 2018 and 2020 spontaneously resolved without intervention. **Conclusions:** This case illustrates the potential for long-term survival in high-risk patients with therapy-related AML developed after cytotoxic treatment for lymphoid malignancies. Haplotransplantation from a semi-identical Human Leukocyte Antigen (HLA) donor proved to be a viable and effective treatment option despite the patient’s age and dual hematologic malignancies.

## 1. Introduction

Chronic lymphocytic leukemia (CLL) is the most common leukemia in adults. Though survival rates are improving with the introduction of new therapeutic agents [1], classical chemoimmunotherapy, such as the FCR regimen (fludarabine, cyclophosphamide, rituximab), remains the gold standard (and has been for more than two decades). Its efficacy in terms of response rates and survival—overall survival (OS), progression-free survival (PFS), and complete remission (CR) rates—was demonstrated in clinical trials and later re-confirmed in younger CLL patients with mutated IgHV status [2,3]; however, the use of FCR in CLL-B has also been associated with a standardized incidence ratio (SIR) for secondary neoplasms of 2.38, significantly higher than in the general population [4]. The risk of therapy-related hematologic neoplasms (t-MN) is increasing, with a median time to the development of t-AML/MDS (therapy-related acute myeloid leukemia/myelodysplastic syndrome) of 2.7 years (range 1.1–7.8 years) and a 5% risk of tAML/MDS after 4.4 years of follow-up as well as a 1.1% risk of t-AML in treated CLL [5,6]. Once a secondary malignancy arises—whether a solid neoplasm, Richter’s transformation, or another hematologic neoplasm—it becomes the primary cause of death. The risk factors implicated in the development of secondary cancers include advanced age at diagnosis, continuous antigenic stimulation, immunodeficiency from the disease and its treatment, disease-related factors, and chemotherapy [6,7]. Prolonged post-treatment cytopenias are an additional risk factor and, in many clinical studies, are the most significant contributor to the development of secondary myeloid neoplasms [6].

Therapy-related AML (t-AML) most commonly presents as acute monoblastic/myelomonoblastic leukemia; however, any subtype of acute leukemia can occur [8,9,10]. In this context, the morphologic and phenotypic subtype of our patient—pDC-AML—has not been previously reported in the literature. Differentiating pDC-AML from a biphenotypic leukemia (myeloid and blastic plasmacytoid dendritic cell neoplasm, BPDCN) based on WHO criteria was challenging but necessary. Typically, when t-AML is diagnosed, a residual CLL clone is also present, but, in our patient, this was not observed, representing a unique feature of the case [7,9].

Response rates in t-AML are much lower than in de novo acute leukemia. Once complete remission is achieved, allogeneic stem cell transplantation is recommended, as it is the only intervention that improves survival, provided comorbidities do not preclude it. To our knowledge, haploidentical transplantation (haplotransplantation) for t-AML secondary to FCR treatment of CLL has not been previously described, although haplotransplants for tAML following chemotherapy for other malignancies have been reported [11].

Differentiating primary AML from therapy-related AML (t-AML) with plasmacytoid features is crucial due to their distinct origins and genetic profiles. While primary AML arises spontaneously and often features balanced translocations, t-AML is linked to prior cytotoxic therapies and frequently involves complex karyotypes, particularly abnormalities in chromosomes 5 and 7, with trisomy 8 being notable. The role of monosomy 8 remains less defined [12].

## 2. Case Presentation

**a. Methods**

To achieve a definitive diagnosis for both diseases, we adhered to the recommended work-up guidelines for these pathologies. A Complete Blood Count (CBC) was performed using Sysmex XT 2000i and XT4000 analyzers (Sysmex Corporation, Japan), with blood collected in tubes containing EDTA as an anticoagulant. Immunophenotyping was carried out on a Beckman Coulter Cytomics FC 500 flow cytometer (argon laser/excitation wavelength at 488 nm), while immunophenotyping of cells from the bone marrow aspirate at the AML diagnosis was performed on the NAVIOS flow cytometer and analyzed with Kaluza software(2.1). Peripheral blood and bone marrow smears were analyzed using May–Grünwald–Giemsa staining. For cytochemistry, MPOX (myeloperoxidase) and PAS (Periodic Acid–Schiff) stains were used. Karyotype examination was conducted by blocking cells with colcemid and analyzing a sufficient number of cells. For real-time PCR, multiplex PCR was utilized.

Coagulation screening tests were performed using Sysmex CS 2000i and CS 2500 coagulation analyzers. Serum glucose and lipid profile parameters were measured using the Architect c8000 system. Immunoglobulins and ASLO were assessed using Cobas 501/6000 through immunoturbidimetry. CRP was measured on the Architect c8000, and ESR was determined on the BD Sedi 40 using a modified Westergren method.

**b. Detailed Case description**

A 57-year-old female nurse presented in April 2010 with odynophagia, fever, chills, bilateral tonsillar hypertrophy, polyadenomegaly, and hepatosplenomegaly (liver 17 cm, spleen 19 cm). A computed tomography scan revealed submandibular, laterocervical, axillary, mediastinal, and retroperitoneal lymphadenopathies (1.6–3.6 cm), some coalescing into masses, compressing nearby organs, particularly in the abdomen. Blood work showed leukocytosis (25.000/mm^3^), with 75% lymphocytes, nuclear shadows on the peripheral smear, hemoglobin of 10.7 g/dL, and platelets at 136,000/mm^3^.

**Figure 1:**
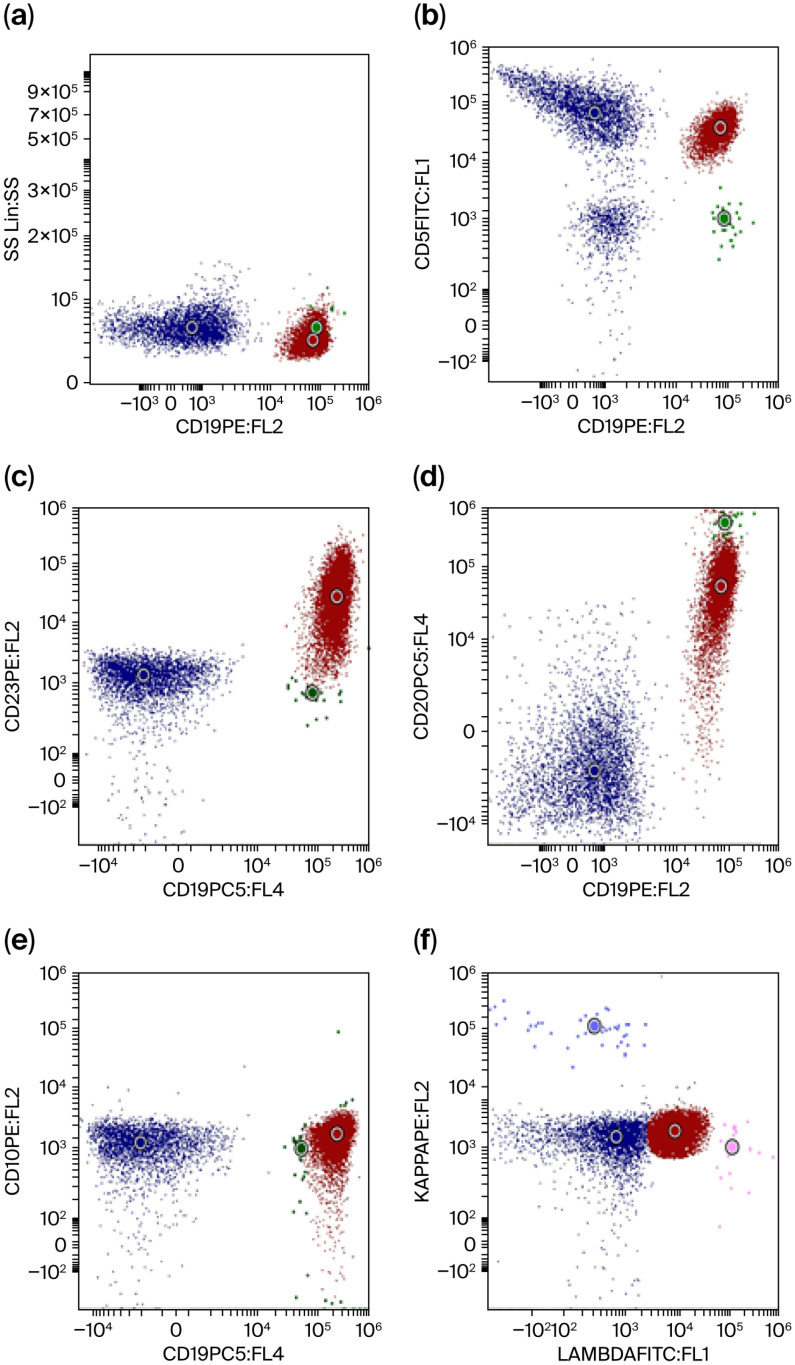
Immunophenotypic characterization of CLL. T cells are blue, B cells are green and further differentiated into Kappa+ (indigo) and Lambda+ (pink), and CLL cells are red. (**a**) Immunophenotypic separation of T cells which are CD19 negative and normal and CLL B cells which are CD19 positive. (**b**) CD5 positivity shown on the CLL B cells. (**c**) CD23 positivity shown on the CLL B cells. (**d**) CD20 downregulated (weakly positive and heterogenous) on CLL B cells. (**e**) CD10 negativity on CLL B cells. (**f**) Positivity for Lambda light chain on CLL B cells.

Immunophenotyping confirmed chronic B-cell lymphocytic leukemia (CLL-B) with a Matutes score of 4 (CD5+, CD20+, CD23+, FMC7-, CD10-, kappa-, lambda+) (Figure 1).

Bone marrow analysis showed 96% infiltration by small mature lymphocytes, consistent with CLLB/SLL. Lymph node biopsy revealed interstitial and nodular infiltration typical of CLL-B.

At the time of diagnosis, testing for p53, 17p deletion, and IgHV status via FISH was unavailable in Romania. Inflammatory markers were normal (ESR = 12 mm/h, fibrinogen = 235 mg/dL, CRP = 2.1 ng/mL). Immunoglobulin levels were slightly reduced (IgG = 700 mg/dL), while IgA and IgM were normal. Negative cultures and ASLO tests excluded infection.

The patient met iwCLL criteria for active disease and required treatment due to progressive lymphadenopathy, hepatosplenomegaly, and symptomatic disease. As a fit patient with no comorbidities, she was eligible for intensive chemoimmunotherapy, and FCR (fludarabine, cyclophosphamide, rituximab) was chosen according to ESMO and NCCN guidelines for fit CLL patients in Romania. She completed six cycles of FCR with complete clinical, biological, imaging, and bone marrow remission confirmed in October 2010. The CT scan showed no pathological adenopathies, no hepatosplenomegaly, and no bone marrow infiltration; however, she developed significant complications related to the chemoimmunotherapy, including severe humoral immune deficiency and prolonged pancytopenia, leading to recurrent infections from 2010 to 2014 in the ENT, urinary, and pulmonary systems. The immune deficiency worsened during therapy but began to recover one year after treatment completion. Grade 3 and 4 cytopenias were recorded, with a minimum neutrophil count of 380/mm^3^, and their recovery occurred only after four months.

Hemoglobin dropped to 6.4 g/dL, recovering within two months, while lymphopenia resolved over seven months. Platelet counts dropped to 12,000/mm^3^, recovering in two months. In January 2017, at the age of 63, approximately 6 years and 9 months after her diagnosis of chronic lymphocytic leukemia (CLL-B) and 6 years and 3 months following the completion of FCR chemoimmunotherapy, the patient was admitted with mucocutaneous pallor, tonsillar hypertrophy, a tonsillar abscess, and general clinical deterioration. Laboratory findings showed a normal leukocyte count (7010/mm^3^), anemia (Hb = 7.5 g/dL), and thrombocytopenia (63,000/mm^3^).

**Figure 2:**
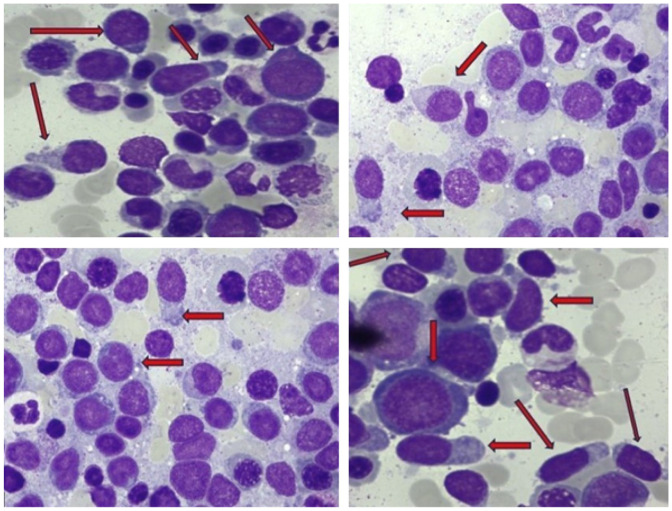
Bone marrow aspirate: MGG stain; 2000× two populations of blasts; one smaller blast with cytoplasmic projections.

Bone marrow aspirate revealed hypercellularity with 30% blasts, which were medium-sized with fine chromatin, 2–4 nucleoli, basophilic cytoplasm without granules, and a second population of smaller blasts with cytoplasmic projections. Triliniar dysplastic changes were noted. Conclusion: hyperplastic bone marrow, appearance suggestive of AML (possibly post-chemotherapy). Blasts were PAS-negative. Peroxidase reaction: 8% positive blasts (Figure 2).

Immunophenotyping analysis of the bone marrow aspirate revealed three distinct populations:

- First Population: 14% of cells, CD45 weakly positive, CD34+, heterogeneous CD117+, weak HLA-DR+, and positive for CD38 and CD25. Approximately half of these cells coexpressed CD22 weakly, indicating they were very young myeloid progenitors.
- Second Population: 10% of cells had weakly positive CD45, HLA-DR+, CD34+/−, CD117−, CD38+, CD123+, and variable CD64. This population was likely oriented toward the monocytic series, beginning to acquire CD64, and included plasmacytoid dendritic cells.
- Monocytic Series: 22% of the cells were positive for CD64, including 4.5% monoblasts, 6–7% promonocytes, and 11% mature monocytes, along with other cell types, indicating a significant monocytic lineage.

**Figure 3:**
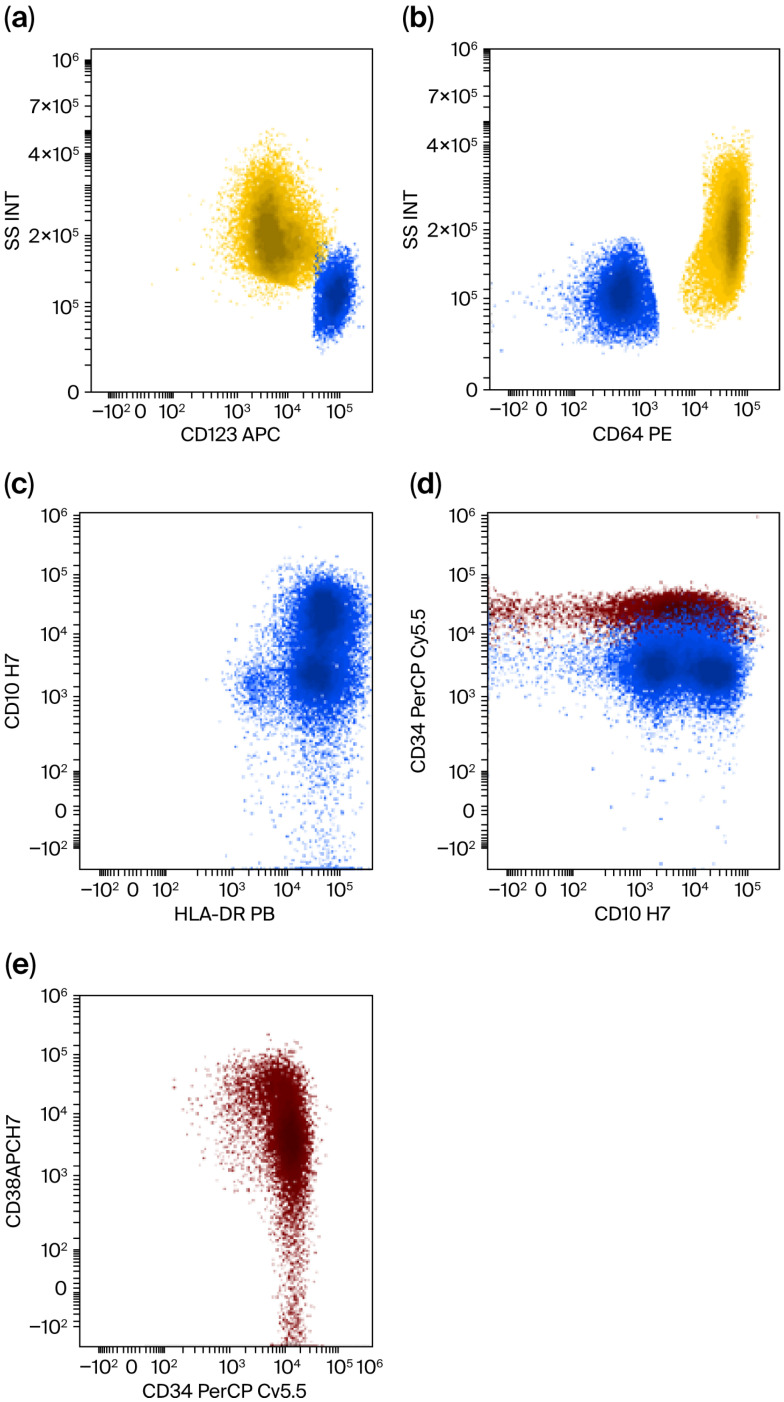
Immunophenotypic diagnosis of therapy-related AML with increased blasts, designated as myelomonocytic AML with increased pDC via immunophenotypic criteria. In red, early progenitors; in yellow, the monocytic line; in blue, pDC. (**a**) pDC with strongly positive CD123 and monocytes with a low CD123. (**b**) Monocytes have very high CD64 while pDCs are negative. (**c**) Atypical CD10 positivity on mature pDC. (**d**) CD10 positivity on progenitors, characteristically found on Multipotent Lymphoid Progenitors. (**e**) Low positivity of CD38 on progenitors, characteristically found on very early progenitors.

Conclusions: The phenotypic profile is suggestive of acute myeloid leukemia (AML) of the myelomonocytic type, associated with plasmacytoid dendritic cells (Figure 3).

The first bone marrow examination revealed bilinear acute leukemia. The bone marrow was hypercellular at about 80%, with diffuse proliferation of blast cells with large, slightly irregular nuclei, 1–3 nucleoli, and variable cytoplasm, and had increased mitosis. Associated with this were blasts with lymphoid characteristics, nucleolated nuclei, and reduced, disorganized cytoplasm, with normocytic precursors and reduced-maturation CD10, which were BCL2-positive in some leukemic blasts.

Subsequent re-examination of the bone marrow revealed 90% hypercellularity, revealing the presence of two distinct populations of blast cells: The first population comprised 50% of bone marrow cellularity, arranged in an interstitial and predominantly nodular pattern. These cells resembled plasmacytoid dendritic cells and expressed CD123 and aberrantly CD10. The second population represented 20% of the cellularity, featuring medium-sized cells with vesicular nuclei and visible nucleoli, expressing CD34 and rarely TdT while being negative for PAX5 and CD3.

A marked reduction in normal hematopoiesis was noted, with mild dysplastic features. Staining for PAX5 and CD23 was negative, indicating no residual infiltration by B-CLL.

The histopathological findings were compatible with bilinear acute leukemia: acute myeloblastic leukemia associated with a proliferation of plasmacytoid dendritic cells, secondary to a myelodysplastic syndrome. The aberrant CD10 expression in plasmacytoid dendritic cells supported the tumoral nature of this proliferation.

Cytogenetic analysis of 21 metaphases demonstrated a mosaic karyotype. While eight metaphases (38%) exhibited a normal 46,XX constitutional karyotype, the remaining 13 cells (62%) revealed an aneuploid clone characterized by monosomy 8. The chromosomal findings are summarized according to the International System for Human Cytogenomic Nomenclature as: 45,XX,−8 [13]/46,XX [8].

In the analyzed sample, the presence of fusion genes was not detected, and both BCR-ABL (minor and major) and FLT3-ITD were negative.

The patient’s diagnosis according to the 2016 WHO classification was t-AML, given the exposure to chemotherapy for a previous malignant hematologic disorder. The difficulty was in classifying the case morphologically and immunophenotypically/immunohistochemically; the differentiation had to be made between pDC-AML and acute biphenotypic leukemia—myeloblastic and blastic plasmacytoid dendritic or acute leukemias of ambiguous lineage. In the absence of a residual CLL clone demonstrated morphologically, immunophenotypically/IHC, this patient’s malignant hematologic disorder was in complete remission with negative minimal residual disease (MRD)- For MRD detection, a DxFLEX flow cytometer equipped with 3 lasers and 8 colors was utilized, achieving a sensitivity of 10^−3^.

The patient underwent five cycles of chemotherapy. The first cycle, a “3 + 7” regimen (Idarubicin 60 mg and Cytarabine 1400 mg total dose per cycle), showed no response. Subsequently, four cycles of high-dose Cytarabine (27 g per cycle) and Idarubicin (60 mg per cycle) were administered, leading to complete remission. These cycles were completed in August 2017, and the treatment was well-tolerated without infections. After the second cycle, the patient obtained complete remission morpho-immunophenotypically and immunohistochemically and remained in remission throughout the treatment. Given the failure of induction therapy, the diagnosis of therapy-related acute myeloid leukemia (t-AML), and the poorer prognosis associated with the plasmacytoid dendritic cell morphophenotype, the patient was recommended for allogeneic hematopoietic stem cell transplantation (HSCT). She had an HCT comorbidity index (CI) score of 1, and, due to the therapeutic urgency, HSCT was indicated.

The donor was selected after discussing options with the patient, who had three siblings with comorbidities preventing them from being suitable donors. As a healthcare worker, the patient opted for a familial haploidentical donor, preferring this option over an unrelated donor transplant due to there being potentially fewer complications associated with haploidentical transplants. The patient’s daughter had given birth to twins, and, although stem cells from the umbilical cords could have been used, the patient opted against this due to the higher risk of leukemia relapse in the absence of graft-versus-host disease (GVHD). Given the lower survival rates in therapy-related acute myeloid leukemia (t-AML) compared to de novo AML and the therapeutic urgency, the decision was made to proceed with haploidentical transplantation. The patient underwent a haploidentical hematopoietic stem cell transplant from her son, utilizing donor peripheral blood stem cells.

The transplant was performed on 11 October 2017, using freshly collected CD34+ cells from the son at a dose of 8.72 × 10^6^/kg. The procedure followed a conditioning regimen of fludarabine (160 mg/m^2^) and melphalan (140 mg/m^2^). Engraftment occurred on day +16 without the need for growth factors. The patient experienced grade 2 mucositis, gastritis, an E. coli infection, and febrile syndrome with negative cultures at another site, but there were no other significant toxicities. GVHD prophylaxis consisted of post-transplant cyclophosphamide (PTCy), administered at 50 mg/kg per day on days +3 and +4. Tacrolimus was administered as monotherapy at a dosage of 0.06 mg/kg/day in two divided doses, with continuous therapeutic drug monitoring for three months. Notably, neither mycophenolate mofetil nor cyclosporine was utilized in this regimen.

From May 2018 to January 2020, the patient showed an increase in leukocytes and lymphocytes. Leukocytosis ranged between 10,500 and 13,000/mm^3^, and lymphocytosis varied between 5400 and 7600/mm^3^. Both the acute and chronic leukemia were in remission, as confirmed through phenotypic and immunohistochemical analysis. While post-transplant lymphoproliferation (polymorphic PTLD) was considered, it was ultimately ruled out. Lymphocyte percentages fluctuated between 35 and 52% without evidence of clonality. Somatic hypermutation of IgHV (DNA) showed polyclonality (absence of B monoclonality), with no detection of del17p or mutations in the TP53 gene. Since January 2020, both leukocytes and lymphocytes have remained within normal limits. The patient was screened for viral infections following the persistent increase in post-transplant lymphocyte counts; tests for Cytomegalovirus and Epstein–Barr Virus were performed and yielded negative results.

Since 2018, the patient developed cortical cataracts in both eyes, which were corrected with new lenses. Additionally, a compressive, non-toxic nodular goiter of the right thyroid lobe was detected, but biopsy results excluded malignant thyroid pathology and chronic lymphocytic thyroiditis.

The patient is currently 15 years post-diagnosis of chronic lymphocytic leukemia (CLL), which was treated with six cycles of FCR, 8 years since the diagnosis of the second malignancy, t-AML, which was treated with five cycles of chemotherapy (1: “3 + 7” and 4 HD-ARA C), and 8 years post-haploidentical stem cell transplantation from her son. She remains in complete remission, with no blasts detected in peripheral blood or bone marrow, morphologically or phenotypically. Chimerism analysis was performed by the transplant center team in accordance with their specific institutional protocols, and stable donor chimerism was maintained throughout the 8-year post-transplant follow-up period. All immunophenotypic evaluations of the patient’s bone marrow aspirate conducted from 2017 to the present (the most recent being in December 2025) have consistently confirmed undetectable minimal residual disease (MRD) for both acute leukemia and chronic lymphocytic leukemia, using international flow cytometric guidelines (Table 1).

## 3. Discussion

Chronic lymphocytic leukemia (CLL) is the most common form of leukemia in adults, accounting for approximately one-third of all leukemia cases and 1% of all cancers, with a 5-year survival rate of 85% [5,6].

At diagnosis, one-third of patients require treatment [7,8].

Treatment decisions are based on several factors, including ESMO and NCCN guidelines, recommendations from CLL working groups, the resources available at the hematology center, comorbidities, and the patient’s preferences. The FCR regimen (fludarabine, cyclophosphamide, and rituximab) has traditionally been the gold standard for treating CLL, with its efficacy validated by multiple independent studies, and has shown a 5-year progression-free survival (PFS) benefit [9]. However, there is a small but significant risk of developing therapy-related acute myeloid leukemia (t-AML) or myelodysplastic syndromes (MDS) following FCR treatment for CLL or other chronic lymphoproliferative disorders. As a result, there is growing interest in moving away from chemoimmunotherapy like FCR in favor of targeted therapies such as Bruton’s tyrosine kinase (BTK) inhibitors, BCL2 inhibitors, and PI3K inhibitors, which can be used alone or in combination, sometimes with anti-CD20 monoclonal antibodies [10,11].

Currently, the FCR regimen (fludarabine, cyclophosphamide, and rituximab) is primarily used in younger patients with chronic lymphocytic leukemia (CLL) who do not have p53 or 17p mutations and who have mutated immunoglobulin heavy variable (IgHV) genes. In these patients, FCR has shown a significant benefit, with a progression-free survival (PFS) of 14.6 years in those with mutated IgHV (IgHV-M) genes compared to just 4.2 years for patients with unmutated IgHV (IgHV-UM) genes [12,13]. Recently, studies have explored combining ibrutinib with FCR, with 6 months of ibrutinib followed by 2 years of maintenance therapy. These studies have shown that 84% of patients achieved negative bone marrow minimal residual disease (MRD) [14,15,16].

Therapy-related myeloid neoplasms (t-MNs), including therapy-related myelodysplastic syndromes (t-MDS) and acute myeloid leukemia (t-AML), are a known complication of chemotherapy, especially in patients with a history of hematologic malignancies. The risk of developing t-MNs depends on several factors, including the type of previous cancer (solid or hematologic), the type of cytotoxic therapy used (e.g., alkylating agents, topoisomerase inhibitors, high-dose chemotherapy, or allogeneic transplantation), the association with radiotherapy, and the time elapsed since the last treatment. Monoblastic leukemias, a subtype of t-MNs, typically develop 3 to 7 years after therapy and are associated with short survival and poor prognosis [15,16].

Patients with a history of hematologic malignancies have the shortest time to develop t-MDS or t-AML, with median times of 46 months, compared to 85 months in those with a history of solid tumors and 109 months in patients with autoimmune disorders. The median overall survival (OS) for these patients is just 14 months, with a 5-year OS rate of only 13.8%. Our case is notable for a survival of 9 years since the onset of tAML, suggesting a more favorable outcome compared to the typical prognosis of this condition.

Treatment options for t-MNs include supportive care, HMA + Venetoclax, an HMA single agent (azacitidine or decitabine), targeted agents (FLT3 inhibitors, IDH inhibitors, etc.), Vyxeos over standard 7 + 3, and, of course, intensive chemotherapy (CLAG, FLAG-Ida, etc.) or allogeneic hematopoietic stem cell transplantation (HSCT), though only 13% of patients receive HSCT due to factors such as age, comorbidities, and disease progression [17,18].

The use of haploidentical stem cell transplantation (haplo-SCT) in older patients with tAML has demonstrated favorable results, with survival outcomes comparable to those achieved with matched unrelated donor (MUD)-HSCT [19,20].

Among the therapy-related neoplasms observed in CLL, solid tumors (especially skin cancers) and t-AML/MDS are the most common, with t-AML occurring in 1% of patients [20]. One study found that, in CLL patients treated with FCR, the median time to develop t-AML/MDS was 2.7 years (ranging from 1.1 to 7.8 years) compared to 1.5 years for solid tumors and 1.1 years for Richter’s transformation [20]. Another found that the median time to tAML/MDS development was 4.47 years in untreated CLL patients compared to 4.19 years in treated patients [21].

Several risk factors have been associated with the development of t-MNs, including advanced age at diagnosis, chronic antigenic stimulation, immunodeficiency, disease-related and therapy-related factors, hypogammaglobulinemia, and the number of chemotherapy cycles. In our patient, diagnosed at 57 years old, several of these factors were present, making her more susceptible to the development of secondary neoplasms. Other potential risk factors include sex, LDH levels, beta-2 microglobulin levels, smoking history, and cytogenetic abnormalities, although some studies have challenged their prognostic significance [4,6,21].

Fludarabine and cyclophosphamide, commonly used in the treatment of CLL, cause DNA damage by inhibiting repair mechanisms and increasing DNA damage in progenitor cells, which leads to prolonged myelosuppression and compromised immune surveillance. The typical cyclophosphamide dose associated with secondary neoplasms is around 11,250 mg/m^2^, though our patient received a lower dose of 7200 mg. Rituximab, which depletes B lymphocytes, is also known to induce long-term neutropenia in 30–52% of patients, further impairing immune function. In our patient, lymphocyte and neutrophil recovery took the longest, with recovery times of 7 and 4 months, respectively [7]. These combined factors contribute to the increased risk of developing therapy-related myeloid neoplasms (t-MNs) and account for the current shift toward alternative treatment strategies for patients undergoing intensive chemotherapy for CLL [22].

Prolonged cytopenias (PC) after treatment is another significant risk factor for the development of tMNs. In fact, up to 70% of CLL patients treated with FCR experience prolonged cytopenias, and those who experience grade 2–4 prolonged cytopenias lasting more than 4 weeks with subsequent resolution have a median time to the onset of t-MNs of 39.9 months, compared to just 17.4 months in those with unresolved cytopenias [6]. In our patient, prolonged pancytopenia lasted for 2 months but ultimately resolved, with t-AML developing 75 months after completing CLL treatment, much longer than the typical timeframe for patients with resolved cytopenias. This prolonged latency further highlights the complexity of t-MN development and the need for ongoing vigilance in managing CLL patients post-treatment [23].

The standardized incidence ratio (SIR) for acute myeloid leukemia (AML) is 2.18 in untreated chronic lymphocytic leukemia (CLL) patients and rises significantly to 9.8 in those receiving treatment [5]. The cumulative incidence of secondary hematologic malignancies after CLL treatment is 2.5%. Between 2000 and 2019, CLL survivors experienced a 158% higher risk of hematologic malignancies, with the gap between the diagnosis of CLL and therapy-related AML (tAML) being approximately 46 months [18,19,20].

t-AML accounts for 5–10% of all AML cases, and, morphologically, it is often challenging to classify as per the French–American–British (FAB) criteria due to its presentation as AML with trilineage dysplasia. The distinction between t-AML and other forms is typically made by the 20% blast threshold [22]. There is ongoing debate as to whether the appearance of tAML is a random occurrence or whether certain individuals may have a genetic predisposition related to drug metabolism and DNA repair. Identifying pre-existing genetic conditions could play a crucial role in selecting therapy, screening, and counseling patients before initiating treatment for the primary disease [23].

In this case, the diagnosis of t-AML was clear based on morphologic findings according to the WHO classification; however, a differential diagnosis was required to distinguish between plasmacytoid dendritic cell (pDC)-AML and acute biphenotypic leukemia (BAL), a form of leukemia that can have myeloblastic and plasmacytoid dendritic cell differentiation. This case, emphasizes not only the complexity of diagnosing t-AML but also the need for personalized treatment strategies considering the genetic background and morphologic challenges associated with secondary leukemia in CLL patients.

The EGIL (European Group for the Immunological Classification of Leukemias) and WHO scoring systems provide diagnostic criteria for BAL wich has very poor prognosis, with a 4-year survival rate of only 8% [24].

Mixed-phenotype acute leukemia (MPAL) comprises a heterogenous group of leukemias that are genetically, immunophenotypically, and clinically diverse, with a mean prevalence of 2.8% of acute leukemia [25,26].

Blastic plasmacytoid dendritic cell neoplasm (BPDCN) is a rare and aggressive hematologic malignancy, accounting for approximately 0.44% of all hematologic cancers. It originates from the precursors of plasmacytoid dendritic cells (pDCs) and is often associated with primary cutaneous involvement, followed by dissemination to the bone marrow as the disease progresses. While it is exceedingly rare for BPDCN to present as leukemia at diagnosis, the leukemic form, known as Blastic Plasmacytoid Dendritic Cell Leukemia (BPDCL), occurs in only about 1% of acute leukemia cases [22].

Diagnosing BPDCL can be challenging due to its clinical, biological, and phenotypic heterogeneity. It often shares overlapping features with other hematologic malignancies, making it difficult to distinguish. A hallmark of BPDCL is the aberrant expression of myeloid and lymphoid antigens, which is frequently observed in affected patients [27].

Given the rarity of BPDCN, treatment strategies are often based on clinical experience. Standard therapies for acute myeloid leukemia (AML) or acute lymphoblastic leukemia (ALL) are frequently employed, but more targeted approaches are emerging: Tagraxofusp (CD123-targeted therapy), Lorvotuzumab (anti-CD56 antibodies), CD123-specific CAR T-cell therapies, bispecific antibodies targeting CD56 and CD123, Nivolumab, venetoclax, and allogeneic hematopoietic stem cell transplantation (HSCT) [28].

Survival outcomes have improved with the use of HSCT: 37.8 months, compared to just 21.1 months for those who did not undergo transplantation [28,29].

Acute myeloid leukemia with plasmacytoid dendritic cell differentiation (pDC-AML) and blastic plasmacytoid dendritic cell neoplasm (BPDCN) are two leukemias characterized by neoplastic proliferation of plasmacytoid dendritic cells (pDCs). pDC-AML represents about 3% of all AML or mixed-phenotype acute leukemia (MPAL) cases, while BPDCN is rarer. In pDC-AML, myeloblasts often show a myelomonocytic immunophenotype with markers such as CD34, CD117, HLA-DR, TdT, and increased CD123 expression. In contrast, BPDCN is characterized by a strong CD56 expression and is more commonly associated with myelodysplastic syndromes (MDS), chronic myelomonocytic leukemia (CMML), and myeloproliferative neoplasms (MPN) [30].

The immunophenotypic profiles of pDC-AML and BPDCN are distinct, with pDC-AML showing higher CD34 and CD117 positivity and BPDCN expressing CD56 and TCL1 at much higher levels. This suggests that the neoplastic pDCs in pDC-AML and BPDCN originate from different subsets of pDC precursors. pDC-AML, which originates from CD34+ blasts, has a poorer prognosis compared to standard AML. In contrast, BPDCN, arising from the CD56+ subset of pDC precursors, is often associated with myeloid neoplasms [31].

At the time of diagnosis in 2017, the WHO 2016 classification of hematologic malignancies was in effect. According to this classification, a definitive diagnosis based solely on immunophenotype was not possible, excluding the patient’s history. Acute leukemia with a mixed phenotype was not considered due to the absence of distinct B/T, T/M, or B/T/M phenotypes. Given the presence of around 15% CD34 + CD38- progenitors and over 20% monocytic lineage, the most immunophenotypically similar diagnosis was AML-M4. While there was an increased population of atypical pDCs (around 10%), they did not exhibit BPDCN features, and this did not define a separate pathological entity. Therefore, the final diagnosis was AML-M4 with increased atypical pDCs. However, the classification has since changed, and, under the WHO 2022 guidelines, the immunophenotypic diagnosis is now pDC-AML, with therapy-related AML with increased pDCs in cases where relevant history is considered [32,33].

**Figure 4:**
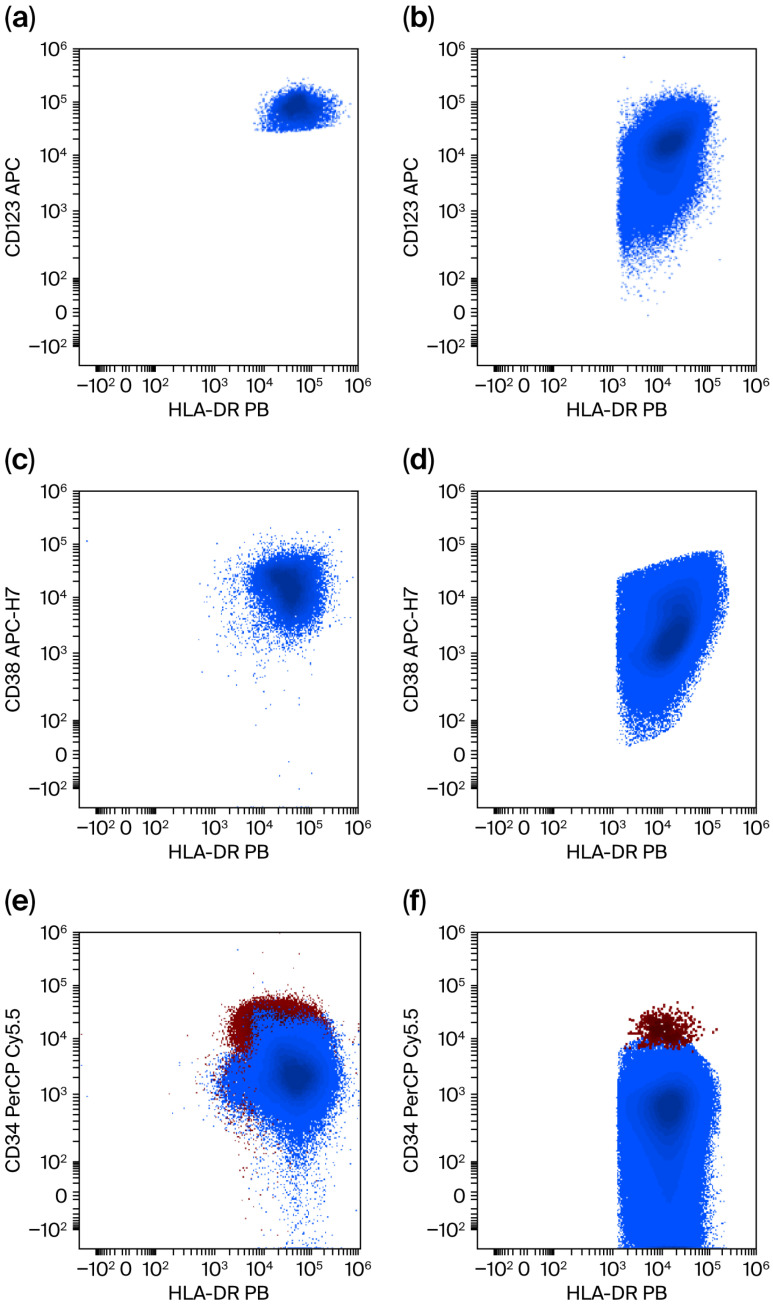

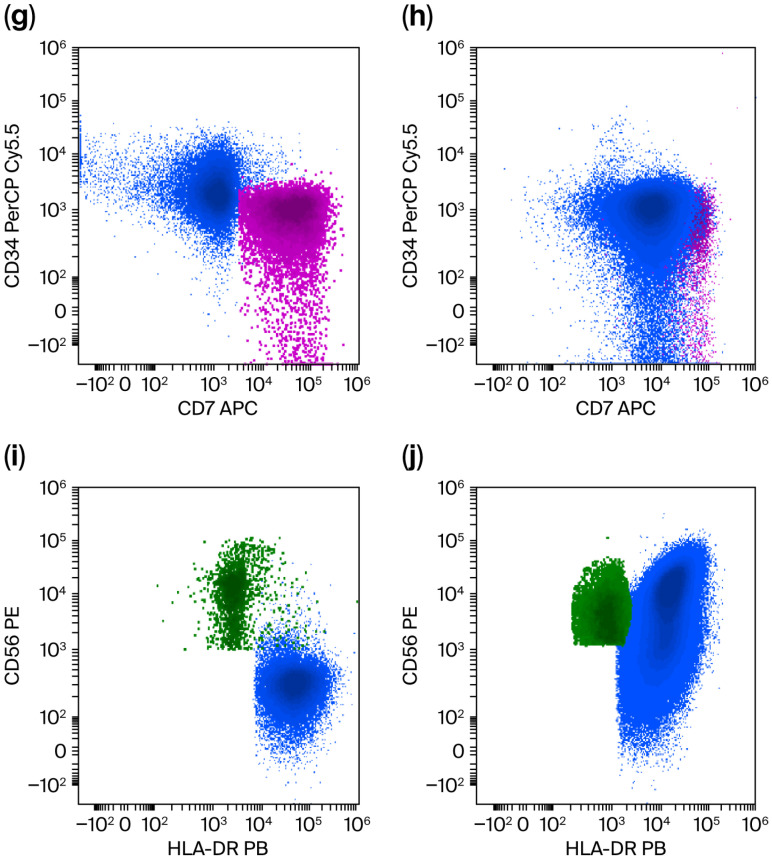
Differences in immunophenotype between normal/reactive pDCs identified in pDC AML and those identified in BPDCN. Blue represents mature pDCs and BPDC, red represents CD34+ progenitors, pink represents T cells, and green represents NK cells. (**a**,**b**) CD123 vs. HLADR. In pDC AML, CD123 is uniformly intensely positive, and HLADR is uniformly positive. In BPDCN, CD123 is heterogeneously positive, indicating downregulation, while HLADR is usually upregulated compared to normal pDCs. (**c**,**d**) CD38 vs. HLADR. In pDC AML, CD38 is uniformly positive, whereas in BPDCN, CD38 is weaker and heterogeneous. (**e**,**f**) CD34 vs. HLADR. In pDC AML, CD34 is positive on a subpopulation of pDCs, and there is a developmental continuum from CD34+ pDC precursors to mature CD34- pDCs. In BPDCN, the precursor where maturation stops is CD34- in the vast majority of cases. (**g**,**h**) CD34 vs. CD7. In pDC AML, similar to CD56 expression, CD7 is negative in the vast majority of cases, while in BPDCN, CD7 is frequently positive. (**i**,**j**) CD56 vs. HLADR. In pDC AML, CD56 is negative in the vast majority of cases, whereas in BPDCN, the vast majority of cases express CD56.

The differential diagnosis between pDC-AML and BPDCN is crucial in this case, especially given the atypical expression of CD10 on mature plasmacytoid dendritic cells (pDCs). Ontogenically, pDCs express CD10 at the progenitor level, so the presence of CD10 on mature pDCs should be interpreted as a sign of dysplasia (Figure 4).

This finding, combined with the observation that 15% of very young progenitors (CD34 + CD38-) are also CD10+, suggests a potential link. A hypothesis arises that the pathological progenitor could be a Multilymphoid Progenitor (MLP), which could give rise to both monocytic cells (which are immunophenotypically normal but elevated in percentage) and CD10+ pDCs [34]. This concept aligns with the observed features and opens a new perspective on the potential origin of the atypical pDCs in this case.

Immunophenotypically, there are several key differences between pDC-AML and BPDCN. Wei Wang et al. describe these differences which were also observed in our patient when compared to a BPDCN patient phenotyped in our clinic who also presented with the cutaneous lesions commonly associated with BPDCN [24,26,27,28].

Upon reviewing the available literature, we could not find any specific significance of monosomy 8 in either de novo or therapy-related acute myeloid leukemia (tAML). The most frequently cited chromosomal abnormalities associated with AML are related to chromosomes 5 and 7, as well as complex karyotypes; however, a relationship with trisomy 8 has been observed in some studies [34,35,36,37].

Although CD303 (BDCA-2) represents a highly specific marker for plasmacytoid dendritic cells and is invaluable in hematologic differential diagnosis, it is not categorized as a general screening marker. TCL1 immunohistochemistry was not available at our institution at the time of diagnosis.

Phenotypic Differential Diagnosis: pDC-AML vs. Reactive pDC Expansion vs. BPDCN/MPAL:

**(1) pDC-AML (AML with pDC Differentiation/Expansion)**

*Arguments for:*

- Clinical context of AML/t-AML: presence of a CD34+/CD117+/HLA-DR+ myeloid blast component associated with a monocytic lineage (high CD64)—consistent with myelomonocytic AML and the previously described subset involving pDC expansion.
- pDC Immunophenotype: pDCs exhibit intense CD123 and HLA-DR expression without the typical BPDCN pattern. The existence of a “pDC precursor continuum” and mature pDCs has been described as a specific feature of pDC-AML in the literature [24].
- Lineage markers: in pDC-AML, CD56 and CD7 are typically negative, in contrast to BPDCN, which is often CD56+ and frequently CD7+ (see Figure 4 and Table 2).

*Arguments against:*

- Aberrant CD10 expression: CD10 expression on mature pDCs may cause confusion with ambiguous lineage entities; however, we interpret this as a sign of dysplasia/transformation within the pDC-AML framework rather than a defining marker for BPDCN.
- Panel limitations: the absence of highly specific pDC markers (e.g., CD303) in the current panel reduces the “granularity” of the differentiation but does not invalidate the diagnosis given the overall clinical and phenotypic picture.

**(2) Reactive pDC/“Normal pDC Expansion” (Non-neoplastic)**

*Arguments for:*

- Clinical Context: theoretically, pDCs can increase reactively during inflammation or infection; the patient presented with a local infection (tonsillar abscess) upon admission.

*Arguments against:*

- Phenotypic Aberrations: the presence of CD10 on mature pDCs, associated with blast populations and trilineage dysplasia, is inconsistent with a purely reactive expansion.
- Histopathology: the bone marrow architecture and tumor burden described histologically (interstitial/nodular pDC-like pattern) support a neoplastic rather than a reactive nature.

**(3) BPDCN (Blastic Plasmacytoid Dendritic Cell Neoplasm)**

*Arguments for:*

- Phenotypic Overlap: the CD123+/pDC phenotype can mimic BPDCN, representing the primary source of diagnostic confusion.

*Arguments against:*

- Lack of a “BPDCN-like” Phenotype: according to Figure 4 and Table 2, BPDCN typically exhibits strong CD56 expression, frequent CD7 positivity, and is usually CD34 negative. In pDC-AML, CD34 may be expressed on a subset, while CD56 is generally negative.
- Clinical Presentation: the patient does not present with the typical cutaneous lesions associated with BPDCN. The global profile (myelomonocytic component + pDC expansion) strongly suggests pDC-AML over BPDCN.
- IHC Limitations: while TCL1 IHC was unavailable, the diagnosis does not rely exclusively on TCL1 when the remaining immunophenotypic pattern is negative for BPDCN.

**(4) MPAL (Mixed-Phenotype Acute Leukemia)**

*Arguments for:*

- Dual Blast Populations: the existence of two distinct blast populations may raise the suspicion of an ambiguous lineage leukemia.

*Arguments against:*

- Absence of Lineage Markers: immunohistochemical examination showed PAX5 and CD3 negativity (lacking definitive B or T-lineage markers). In the absence of specific lineage antigens, MPAL criteria are not met.
- Lineage Commitment: the monocytic component (high CD64) and CD34+/CD117+ myeloid blasts further support myelomonocytic AML with pDC expansion rather than MPAL.

**(5) “BPDCN/MPAL Overlap” (BPDCN-like MPAL/Borderline Entities)**

*Arguments for:*

- Literature Comparison: the patient’s profile is comparable to cases cited in the literature as “AML mimicking BPDCN.”

*Arguments against:*

- Primary Driver: the “driver” is an AML/t-AML with a monocytic component and myeloid blasts. The pDC profile lacks the BPDCN signature (CD56/CD7), and B/T-lineage markers are negative; therefore, a true BPDCN/MPAL overlap is not supported.

Therapy-related acute myeloid leukemia (t-AML) with plasmacytoid dendritic cell (pDC) differentiation is a rare and complex condition. While specific case reports are limited, several studies have explored the characteristics and treatment outcomes of such cases.

A study by Wei Wang et al. analyzed 53 cases of pDC-AML, representing about 3% of all AML cases. The research characterized their immunophenotype and genetic profiles, highlighting the distinct features of pDC-AML compared to blastic plasmacytoid dendritic cell neoplasm (BPDCN) [24].

The development of t-DC-AML in our patient precisely mirrors the long-term safety signals identified in the E2997 Intergroup Study [38]. Our case exemplifies the clinical trade-off described by Smith: while the potent synergy of fludarabine and cyclophosphamide effectively achieves deep remission in CLL, it concurrently increases the cumulative risk of therapy-related myeloid neoplasms by fourfold compared to monotherapy.

Another study, by Xiao et al., described a subset of AMLs displaying expansion of mature pDCs, emphasizing the importance of recognizing this subset for accurate diagnosis and treatment planning [39].

Additionally, a case report published in 2023 detailed a 57-year-old female presenting with cervical lymphadenopathy and skin rashes during the COVID-19 pandemic following multiple types of chemotherapy. This case underscores the potential for pDC proliferation in patients with a history of chemotherapy [40].

These studies and case reports provide valuable insights into the presentation, diagnosis, and treatment of t-AML with pDC differentiation, highlighting the complexity and rarity of this condition.

While the case presented in this manuscript achieved long-term remission, comparing it to similar cases in the literature helps contextualize this outcome. Therapy-related acute myeloid leukemia (tAML) with plasmacytoid dendritic cell (pDC) differentiation, although rare, is typically associated with poor prognosis; however, some studies have reported instances of long-term remission, particularly in patients who received aggressive chemotherapy followed by hematopoietic stem cell transplantation (HSCT) [39].

Studies by Chang et al. and Sanz et al. underline the potential for positive outcomes even in complex tAML cases, especially when minimal residual disease is well-controlled and stem cell transplantation is administered promptly. This case adds to the growing body of evidence showing that, despite the high-risk nature of t-AML with pDC differentiation, long-term remission is achievable with appropriate treatment strategies [41,42].

The median survival for patients with t-MDS/tAML receiving standard chemotherapy is 7–12 months, with survival outcomes improving significantly only after the performance of HSCT. A CIBMTR analysis of alloHCT outcomes showed a 5-year overall survival (OS) rate of 22% and disease-free survival (DFS) rate of 21%. Age remains a negative prognostic factor for transplant outcomes, with studies suggesting a significant decline in survival outcomes for patients over 41 years (EMBT), 36 years (CIBMTR), and 50 years (Japanese transplant group). In this case, the patient was 63 years old at the time of transplant, but studies encourage the use of haploidentical stem cell transplantation (haplo-SCT) in elderly patients, as age alone should not be considered a formal barrier. FluMel conditioning, which was used in this case, has been shown to result in lower relapse rates compared to Flu-Busulfan conditioning [43,44].

Several studies highlight the favorable outcomes of haplo-SCT compared to matched unrelated donor (MUD) transplantation. For example, post-transplant cyclophosphamide has been shown to reduce the effects of graft-versus-host disease (GVHD), which may contribute to improved outcomes in haplo-SCT, especially in patients with positive MRD [45].

Recent studies found that OS and relapse-free survival (RFS) rates were significantly lower in patients with therapy-related myeloid neoplasms (tMN) compared to those with de novo disease (31% vs. 44% and 27% vs. 41%, respectively) [46]. Nonetheless, haplo-HSCT may offer similar survival outcomes to MUD-HSCT in the treatment of acute leukemia. Yin-Che Wang’s study found no substantial differences in OS or leukemia-free survival (LFS) between patients receiving haploHSCT and those receiving MUD-HSCT [47].

A study by Wall et al. reported promising survival outcomes following haplo-HSCT in elderly patients with therapy-related AML, showing that age is not a contraindication for successful stem cell transplantation when managed appropriately [48].

A report by Jiang et al. analyzed the role of MRD monitoring in post-transplant patients with t-AML, revealing that aggressive post-transplant strategies can significantly improve long-term remission in patients with minimal residual disease at the time of transplant [49].

Bai et al. demonstrated that combining innovative conditioning regimens with haploidentical transplantation improved 3-year disease-free survival rates in t-AML, particularly when MRD was effectively managed prior to the procedure [50].

These findings underscore the growing evidence supporting the use of haploidentical stem cell transplantation for patients with therapy-related acute leukemia, particularly for those of older age or with minimal residual disease, where traditional allogeneic options may be limited, even in challenging t-AML cases with plasmacytoid dendritic cell differentiation.

This case adds valuable insights to the existing knowledge on therapy-related acute myeloid leukemia (t-AML) and haploidentical stem cell transplantation (haplo-HSCT). It highlights the potential for long-term remission in t-AML patients following haploHSCT, especially in high-risk cases where traditional therapies have failed. The successful outcome in this patient despite the presence of plasmacytoid dendritic cells, a marker associated with poorer survival, demonstrates the efficacy of haplo-HSCT as a viable treatment option for secondary AML. This case also sheds light on the use of haplo-HSCT as a therapeutic alternative to unrelated donor transplants, showcasing the feasibility and potential advantages of familial donor sources.

Additionally, the exclusion of post-transplant lymphoproliferative disorder (PTLD) emphasizes the importance of careful post-transplant monitoring. Lastly, the patient’s post-transplant surveillance, including the presence of polyclonal lymphocytosis and the absence of monoclonality, contributes to our understanding of how subtle signs can distinguish remission from relapse in haplo-HSCT recipients. Between May 2018 and January 2020, the patient presented with persistent leukocytosis and lymphocytosis (WBC 10.5–13 × 10^9^/L; lymphocytes 5.4–7.6 × 10^9^/L; 35–52%), despite being in complete morphological and phenotypic remission for both primary malignancies. Given the history of Chronic Lymphocytic Leukemia (CLL) and post-Hematopoietic Stem Cell Transplantation (HSCT) status, the clinical priority was to differentiate between benign/polyclonal lymphocytosis and Post-Transplant Lymphoproliferative Disorder (PTLD) or clonal relapse of the CLL.

Evidence Supporting Polyclonal Lymphocytosis:

- Absence of B-cell clonality: repeated immunophenotypic monitoring showed no evidence of a re-emerging monoclonal B-cell population compatible with CLL.
- Polyclonal IGHV (DNA): analysis of somatic hypermutation patterns demonstrated IGHV polyclonality (absence of B-cell monoclonality), a finding that contradicts both monomorphic PTLD and CLL relapse.
- Negative Viral Status: viral screening for EBV and CMV remained negative, significantly reducing the probability of an EBV-driven PTLD.
- Benign Clinical Course: the lymphocytosis resolved spontaneously without medical intervention. Since January 2020, both white blood cell and lymphocyte counts have remained within normal ranges—a progression highly atypical for PTLD or neoplastic relapse.

We explored potential links between our case and patients included in clinical trials or long-term follow-up cohorts treated with frontline FCR. The clinical course of our patient, marked by the development of plasmacytoid dendritic cell acute myeloid leukemia (pDC-AML) following intensive chemoimmunotherapy, underscores the long-term hematologic vulnerabilities and genomic instability described in the CLL8 trial, illustrating how iatrogenic DNA damage to hematopoietic stem cells may culminate in high-grade myeloid transformation [51]. The development of pDC-AML in our patient reflects the late-hit leukemogenic model described by Benjamin et al., in which cumulative DNA damage induced by frontline FCR therapy manifests as a secondary myeloid neoplasm years after the initial treatment. This association is particularly evident in the latency period of our case, consistent with the 5.1% cumulative incidence observed in the MD Anderson cohort at 10 years. Our findings suggest that the intense selective pressure of fludarabine-based regimens not only targets the CLL clone but also predisposes the myeloid progenitor compartment to leukemogenic events [4]. Studies dedicated to secondary myeloid neoplasms (t-MDS/AML) following fludarabine-based therapy (FC/FCR) confirm the significance of our case. One of these studies is the previously mentioned work by Smith et al. [38]. The morphologic findings in our patient, particularly the marked trilineage dysplasia and the interstitial/nodular distribution of the pDC expansion, are highly characteristic of the therapy-related myeloid landscape described by Zhou et al. [52]. Their study of FCR-treated CLL patients demonstrated that such dysplastic features represent a hallmark of iatrogenic myeloid transformation rather than reactive processes.

As highlighted by recent large-scale analyses conducted by Laribi et al. [7], the risk of myeloid neoplasms related to FCR therapy is significant, with a cumulative incidence that continues to increase even after completion of treatment. Our case of PDC-AML serves as a clinical illustration of this late toxicity. Furthermore, the aggressive nature and unfavorable survival outcomes associated with t-MN reported in the literature reinforce the severity of the diagnosis in our case and support the need for a curative-oriented therapeutic strategy, with intensive chemotherapy targeting the myeloid clone.

Real-world and population-based studies similarly converge on this conclusion. The diagnosis of pDC-AML in our patient must be interpreted within the broader epidemiological context described by van der Straten et al. [53]. Their 30-year population-based study confirms that patients with CLL face an almost twofold increased risk of developing secondary primary malignancies compared with the general population, with a particularly elevated risk for acute myeloid leukemia (SIR 2.76). This baseline vulnerability, combined with the subsequent iatrogenic insult of FCR therapy, likely created the multi-hit genomic environment necessary for the emergence of the pDC-AML clone.

The clinical course of our patient is consistent with the findings of Fürstenau et al. [54], who demonstrated a significantly higher cumulative incidence of secondary malignancies in treated CLL patients compared with untreated cohorts. In our case, these data support the hypothesis that FCR therapy acted as the primary driver of subsequent myeloid transformation (pDC-AML), exacerbating the underlying genomic instability inherent to CLL patients, as evidenced by data from the GCLLSG registry.

The clinical course of our patient is representative of the long-term risks associated with frontline FCR therapy in Central Europe, as recently reported by Kösa et al. [6]. The occurrence of pDC-AML approximately 75 months after FCR treatment closely aligns with the median latency described in their multicenter study. This case provides clinically relevant validation of their findings, indicating that secondary myeloid neoplasms constitute a severe late complication that may offset the initial success of CLL eradication, thereby supporting a clinical shift toward non-genotoxic targeted therapies. The long-term analysis of the GCLLSG registry by Kutsch et al. provides clear evidence of late-onset risks associated with frontline FCR therapy [55]. Our case of secondary acute myeloid leukemia (pDC-AML) exemplifies the clinical challenge highlighted by the German group, namely the development of aggressive secondary myeloid neoplasms long after successful control of the primary disease. These findings reinforce the clinical consensus that the genomic toxicity of purine analogue-based regimens persists as a driver of late mortality, supporting the paradigm shift toward targeted, non-cytotoxic therapies in CLL management.

Characteristics of patients that developed AML after frontline FCR (5 pc/234 pc MDACC January 2004–March 2012 [4]) compared with our case can be seen in Table 3. 

Compared to the five cases of AML following CLL treated with FCR described by Benjamin et al [4]., we observed the following in our case:

1. The age of patients with CLL who later developed AML was relatively young, with a mean age of 59 years. All patients were under 66 years old, even though CLL is typically a disease of the elderly. One would expect that this older population, with higher genetic instability, would more frequently develop a secondary hematologic malignancy. Clonal hematopoiesis of undetermined potential (CHIP) is common in older individuals and is associated with an increased risk of therapy-related myeloid neoplasms due to the advanced age of CLL patients. AML was typically diagnosed at a younger age (below 60), while MDS and non-melanoma skin cancer (NMSC) were mostly diagnosed in patients over 70 years in three countries. The incidence of AML developing during the course of CLL or after CLL treatment is 1.7% and 1%, respectively, accounting for 10.9% and 8.3% of all secondary malignancies.

2. Only our case involved a female patient, even though it has been shown that, in women over 60, the risk of secondary neoplasms in general is higher. This may be due to greater myelotoxicity from chemoimmunotherapy, possibly explained by gender differences in pharmacodynamics. Elderly female patients were found to have slower rituximab clearance.

3. Most patients were at Rai stage 2, like our patient, but t-AML can also appear in stage 1, as shown in the table. Our patient had the lowest lymphocyte count at diagnosis.

4. All patients had cytogenetic abnormalities, either diploidy, complex, or abnormal karyotypes. Three patients had trisomy 12. Only our patient presented with monosomy 8 and one patient with del(11q), and these two were the only long-term survivors, living more than 8 years.

5. Among all patients, only our case received the lowest dose of standard FCR chemoimmunotherapy, while the others received more aggressive regimens—generally FCR3, FCMR, or FCR with alemtuzumab—which may plausibly explain the better survival in our patient’s case. FCR3 resulted in similar response rates, time to progression (TTP), and overall survival (OS) compared to a historical cohort treated with FCR. However, FCR3 was associated with an increased incidence of t-MDS/AML [4].

6. Except for one patient, all received six cycles of therapy and achieved complete remission after treatment.

7. In our patient and the other long-term survivor with high OS, the latency period from the end of FCR treatment to the appearance of AML was the longest—75 months and 96 months, respectively. For the rest of the patients, the latency period was shorter—4 years, or even as little as 18 months in the patient who received alemtuzumab.

In the other three cases identified in the literature, we found the following differences compared with our patient (Table 4):

## 4. Conclusions

In conclusion, this case demonstrates the complexity and challenges in managing patients with chronic lymphocytic leukemia (CLL) who develop therapy-related acute myeloid leukemia (t-AML) with plasmacytoid dendritic cells, a condition that requires careful differentiation from blastic plasmacytoid dendritic cell neoplasms (BPDCN) [44,45]. The patient’s favorable outcome, with a remarkable overall survival of 15 years following the initial hematologic malignancy diagnosis and 8 years after haploidentical stem cell transplantation for t-pDCAML, underscores the importance of early diagnosis, intensive chemotherapy, and the role of multidisciplinary collaboration in achieving long-term survival.

## Figures and Tables

**Table 1:** Timeline of disease transformation and treatment milestones.

April 2010	Chronic lymphocytic leukemia (CLL)	6 cycles FCR Fludarabine 25 mg/m^2^ IV on days 1–3 Cyclophosphamide 250 mg/m^2^ on days 1–3 Rituximab 375 mg/m^2^ on day 1 of cycle 1, 500 mg/m^2^ on day 1 of cycles 2–6
October 2010	The last chemotherapy for CLL	Complete remission
2010–2016	Severe humoral immune deficiency Prolonged pancytopenia Recurrent infections	Acute right pyelonephritis (October 2010) Shingles (November 2010) Acute enterocolitis (January 2011) Sinusitis and viral–bacterial pneumonia (March 2011) Cryptic tonsillitis (2012) Bilateral maxillary sinusitis (2013) Urinary tract infection (2014) Pharyngotonsillitis (2016)
January 2017	Therapy-related acute myeloid leukemia (t-AML) with plasmacytoid dendritic cells (pDC-AML)	Cycle 1: “3 + 7”: idarubicin 12 mg/m^2^ on days 1–3 (60 mg total dose) + cytarabine 100 mg/m^2^ on days 1–7 (1400 mg total dose)—no response after cycle 1 Cycle 2: HiDAC + IDA: 3 g/m^2^ q12 h—on days 1–3 (27 g total dose) + 12 mg/m^2^ on days 1–3 (60 mg total dose)—complete response; MRD negative Cycles 3–5 identical to cycle 2
August 2017	The last chemotherapy for t-AML	Complete response; MRD negative
October 2017	Haploidentical stem cell transplantation from son	Conditioning with fludarabine 160 mg/m^2^ and melphalan 140 mg/m^2^ infusion of CD34+ cells at a dose of 8.72 × 10^6^/kg
December 2017–March 2018	GVHD prophylaxis	PT Cy 50 mg/kg per day on days +3 and +4. Tacrolimus 0.06 mg/kg/day in two divided doses three months.
May 2018–January 2022	Leukocytosis and lymphocytosis	Post-transplant polyclonal lymphocytosis Monitoring to exclude post-transplant lymphoproliferation Somatic hypermutation of IgHV (DNA) demonstrated the presence of polyclonality (absence of B monoclonality), del17p, and mutations in the TP53 gene were absent
2018–Present Non-Hematologic Complications	Cortical cataracts Compressive, non-toxic nodular goiter	Corrected with new lenses Biopsy excluding malignant thyroid pathology
Alive	15 years following the initial hematologic malignancy diagnosis and 9 years after haploidentical stem cell transplantation for t-pDC-AML	CLL + pDC-AML post-haploidentical transplant: sustained complete remission

**Table 2:** Summarized immunophenotypic profile.

	BPDCN Blasts	Normal PDCs	Monocytic Lineage
**CD34**	−	−/small subpopulation +	−/small subpopulation +
**CD45**	+ low	+	+
**HLADR**	++/+++	+++	−/+
**CD117**	−/+ low	−/small subpopulation +	−/small subpopulation +
**CD64**	−	−	+++
**CD123**	+++ het *	+++ hom **	−/+ low/+ mid
**CD4**	+	+	+
**CD38**	+ het *	++	+
**CD7**	+ het *	−	−
**CD10**	−/+	−	−
**CD56**	++	−	−

* heterogenous; ** homogenous; BPDCN is excluded if any lineage-specific antigen is positive (e.g., CD19 for B celll differentiation, cyCD3 for T cell differentiation, CD64 (very high) for monocytic differentiation.

**Table 3:** Frontline FCR and subsequent AML: MDACC cohort (5/234, 2004–2012), in context of our case, standard FCR = FCR; FCR with thrice higher rituximab per cycle = FCR3; FCR with mitoxantrone = FCMR; FCR with alemtuzumab = CFAR.

No. pc	1	2	3	4	5	Our Case
Age at time of AML (year)	58	61	44	63	66	63
Sex	M	M	M	M	M	F
Rai stage	2	1	3	2	2	2
Absolute lymphocyte count	96	80	126	324	154	18
LDH	1030	505	490	797	2182	375
B2M	9.6	4.8	5.1	2.9	10	3
IgVH mutation status	NA	NA	MU	MU	NA	NA
Cytogenetics	diploid	abnormal	complex	diploid	diploid	abnormal
FISH	T12	neg	del11q	T12	T12	monosomy 8
Treatment	FCR3	FCR3	FCR3	FCMR	CFAR	FCR
No. of cycles	6	6	6	6	5	6
Response	RC	RC	RC	RC	RC	RC
Latency from treatment (month)	40	53	93	46	18	75
Time to last follow-up from Tx (year)	3.6	4.5	8	3.6	1.5	8
Status at last follow-up	dead	dead	alive	dead	dead	alive

**Table 4:** Clinical characteristics of AML secondary to CLL: literature review and case comparison.

No.	Age/Sex	AML Type	Latency from Treatment (Month)	CLL Treatment	Cytogenetic	Transplant	Result	Status at Last Follow-Up	Reference
1	51/M	t-AML monocytic	12	FCR	t(8;16)(p11,p13)	N/A	N/A	N/A	Gajendra, S. et al., 2023 [8]
2	55/M	AML	48	CLL untreated	del(7q)	Allo-HSCT (unrelated)	CR	Alive	DeFilipp, Z. et al., 2012 [20]
3	60/F	t-AML myelomonocytic	72	FCR	45–47,XX, del(5)(q13q33), trisomy 8, trisomy 20, monosomy21, mar[cp12]/46,XX	N/A	CR	Dead in consolidation	Wach et al., 2017 [56]
4	63/F	t-AML monocytic pcAML	75	FCR	monosomy 8	Haploidentical transplant	CR	Alive	Our case

Allo-HSCT: allogeneic hematopoietic stem cell transplantation; N/A: not available.

## Data Availability

The data that supports the findings of this case report is available from the corresponding author upon reasonable request. Due to privacy or ethical restrictions, some data may not be made publicly available to protect patient confidentiality. Data sharing is in compliance with the institutional and national ethical guidelines.

## Abbreviations

The following abbreviations are used in this manuscript:

CLL	Chronic Lymphocytic Leukemia
FCR	Fludarabine, Cyclophosphamide, and Rituximab
t-MN	Therapy-Related Myeloid Neoplasms
t-AML	Therapy-Related Acute Myeloid Leukemia
pDC-AML	Acute Myelomonocytic Leukemia with Plasmacytoid Dendritic Cells
BPDCN	Blastic Plasmacytoid Dendritic Cell Neoplasm
GVHD	Graft-Versus-Host Disease
HSCT	Hematopoietic Stem Cell Transplantation
HLA	Human Leukocyte Antigen
MRD	Minimal Residual Disease
SIR	Standardized Incidence Ratio
OS	Overall Survival
PFS	Progression-Free Survival
CR	Complete Remission
FAB	French–American–British Classification
EGIL	European Group for the Immunological Characterization of Leukemias
MPAL	Mixed-Phenotype Acute Leukemia
HD-ARA C	High-Dose Cytarabine
PTCy	Post-Transplant Cyclophosphamide
FLT3ITD	FMS-Like Tyrosine Kinase 3 Internal Tandem Duplication
iwCLL	International Workshop on Chronic Lymphocytic Leukemia
CHOP-R	Cyclophosphamide, Hydroxydaunorubicin, Oncovin, Prednisone with Rituximab
CVP-R	Cyclophosphamide, Vincristine, Prednisone with Rituximab
PTLD	Post-Transplant Lymphoproliferative Disorder
BCR-ABL	Breakpoint Cluster Region-Abelson
CD	Cluster of Differentiation
CIBMTR	Center for International Blood and Marrow Transplant Research
NCCN	National Comprehensive Cancer Network
WHO	World Health Organization
MDS	Myelodysplastic Syndrome
AML-M4	Acute Myelomonocytic Leukemia
HCT-CI	Hematopoietic Cell Transplantation Comorbidity Index
TAG	Tagraxofusp
CAR	Chimeric Antigen Receptor
ESMO	European Society for Medical Oncology
FDA	Food and Drug Administration
NK	Natural Killer (Cells)
